# The Solubility of Hen Lysozyme in Ethylammonium Nitrate/H_2_O Mixtures and a Novel Approach to Protein Crystallization

**DOI:** 10.3390/molecules15020793

**Published:** 2010-02-04

**Authors:** Nolene Byrne, C. Austen Angell

**Affiliations:** Department of Chemistry and Biochemistry, Arizona State University, Tempe 85287, AZ, USA; E-Mail: austenangell@gmail.com (C.A.A.)

**Keywords:** protic ionic liquids, protein crystallization, solubility and protein stability

## Abstract

We report on the solubility of hen lysozyme (HEWL) in aqueous ethylammonium nitrate (EAN) as a function of water content. We find the solubility behavior to be complex, exhibiting both a maximum (400 mg/mL) at very high EAN content) and a minimum at intermediate EAN content. We exploit this solubility profile in a novel approach to generating crystals of hydrophilic proteins, based on *rehydration* of a high concentration protein solution. We describe the production of crystals of X-ray diffraction quality. Two related ionic liquid solvent systems, with the same solubility profiles but different effective pH characteristics, are identified for future evaluation.

## 1. Introduction

The solubility of proteins plays an important role in a range of industrial processes including those of the pharmaceutical and food industries [1,2,3,4]. Protein purification also relies on a knowledge of the solubility of the protein in the chosen process buffer. The solubility of a given protein in a given solvent depends largely on the content and distribution of the hydrophilic and hydrophobic amino acids on the protein’s surface. Other factors contribute to protein solubility, most notably pH. The minimum solubility for a given protein in an aqueous solution occurs near its isoelectric pH [5,6,7,8,9,10]. This is due largely to a lack of electrostatic repulsions at this pH between the protein and solvent [9,10]. Since the solvent modulates the electrostatic repulsions between the protein molecules, protein solubility functions can be rather different in different solvents [2,3].

Solubility is a key parameter for protein crystallization. Protein crystallization, in addition to its usual role in revealing the 3-D structure of the protein and providing insight into the protein’s biological function, presents a possible and potentially valuable fast track route to protein purification [11] (and also storage) if it can be made facile. Producing protein crystals, however, is usually very difficult, and in the majority of cases has not been achieved. Many protein crystallization techniques have been developed but they are mostly based on the same underlying concept/strategy. The strategy involves the dehydration of the solution containing the protein so as to create a supersaturated solution [10,11]. The protein-crystallizing solution is often rather complex, containing, in addition to the stabilizing buffer, various components which, in one way or another, aid in the nucleation process (“precipitating additives”). The product is often slow to form, poor in yield, and low in quality (the latter beng judged by the sharpness of the X-ray diffraction spots). Due to the statistical nature of the nucleation process, reproducibility of results is frequently a problem. Proteins are, in general, more difficult to crystallize then other small molecules or inorganic compounds. However the same two steps, first a nucleation and then a growth process, are common to all. The quality of the crystal is highly dependent on the occurrence of a quality nucleus and an orderly growth.

Recently, ionic liquids (ILs; ambient temperature liquids consisting entirely of ions) have been found effective as additives in the standard protein crystallization protocol. In each of the reported studies [12,13,14,15] different ionic liquids have been used at varying concentrations but in each case the IL was used as a precipitating additive present in both the solution droplet and the reservoir. In most cases increased crystallization rates and, in some cases larger crystals, were produced by the IL addition. ILs have many applications [16,17,18,19,20], one of special interest to the present study being their use as preserving media for biomolecules. The recently demonstrated ability of ILs to dissolve large amounts of protein, up to 400 mg/mL, while protecting them against degradation by aggregation or hydrolysis [21], is very relevant to the possibility of using them in new approaches to the protein crystallization challenge. It is with this challenge that the present article is concerned. We will use the unusual solubility profile presented by proteins in hydrated *protic* ionic liquid (PIL) media as the basis for a novel approach to protein crystal generation. This paper then consists of two parts, one related to establishing the protein solubility profile in these solvents, and a second devoted to exploiting the solubility profile to facilitate protein crystal formation by a new route. We have chosen the much-studied hen egg white lysozyme (HEWL) as the target protein, and the likewise well-known PIL, ethyalmmonium nitrate (EAN), as the solvent component.

## 2. Results and Discussion

### 2.1. Solubility

**Figure 1:**
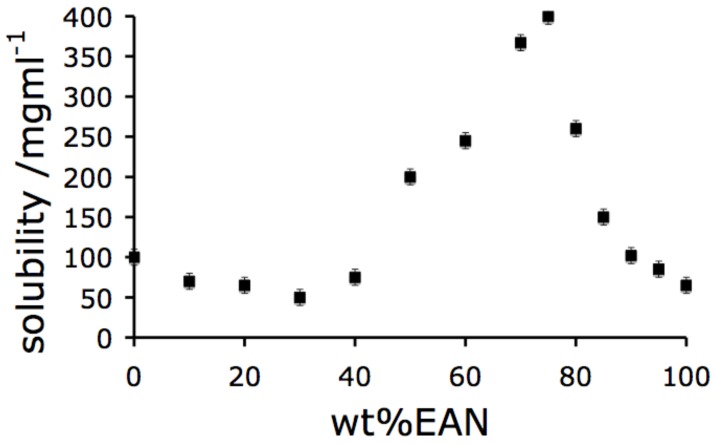
Solubility profile for Hen lysozyme in EAN/water mixtures, as determined using light scattering assessments of the solution state.

The phase diagram for the binary system, EAN + water, is very simple. It features only a simple eutectic at -36 ºC at a composition of 30 mole % EAN. The ambient temperature solubility of HEWL in EAN-H_2_O solutions of various compositions is shown in Figure 1. The solubility of HEWL in this binary mixture is complex. At the anhydrous limit, the solubility of HEWL is 100 mg/mL, comparable with that in pure water. Upon addition of water to the anhydrous EAN, the solubility increases (as noted previously [22]) until a maximum at a remarkable 400 mg/mL (four time larger than the solubility of HEWL in pure water) is reached at a solvent composition of 25 wt % water. This corresponds to 33 mol% EAN, or one water molecule for each ion. At a protein content of 400 mg/mL the solution is approximately 30 vol. % protein.

The protein concentration seen in Figure 1 is far higher than any solubility reported for HEWL organic solutions, where the maximum value observed has been only ~10 mg/mL [23]. Water increases HEWL solubility in organic solvent + water systems, but the authors are not aware of concentrations as high as 400 mg/mL being reported previously. The reason why such an extraordinary solubility for a biomolecule should be found in a medium so rich in ions is a question that deserves detailed discussion. However our attention here is on using the solubility profile to design a novel approach to protein crystallization. Thus we postpone the discussion of the solubility itself to later works when a broader empirical base will be available. That the high solubility is not unique to EAN-H_2_O solvents will be shown later in the paper. Thus it is likely to have some generic explanation, elements of which may well lie in a conjecture concerning early earth biology that we have put forward elsewhere (ref. [26]).

Beyond the maximum, the solubility of HEWL decreases to an interesting minimum at a water loading of 70 wt % which we will take to advantage in the protein crystallization studies to be described below. At the minimum, the solubility has dropped by almost an order of magnitude, to 45 mg/mL, nine times less then the maximum solubility observed. Beyond the minimum, the solubility again increases, rising continuously to its well -known value of ~100 mg/mL in pure water.

In the latter high concentration range, loss of biologically activity is observed within 24 h, according to our studies. As previously mentioned, we have shown that 200 mg/mL HEWL dissolved in an 80 wt % EAN solution remains in a native state for periods of at least three years [21]. The complex solubility behaviour of HEWL in EAN/water mixtures is also seen in two other protic ionic liquid solutions discussed later, and must have some generic explanation, which we do not discuss further here. The low solubility observed in the anhydrous EAN solution is likely a consequence of the absence of the hydration layer - which is particularly important for hydrophilic proteins such as HEWL.

### 2.2. Stability

Of paramount importance to any crystallization process aimed at producing crystals of complex molecules suitable for structural analysis, is that the substance being crystallized remain stable while the drawn-out process of nucleation and growth of the desired crystal is in progress. One of the advantages of using ionic liquids as the solution medium in the generation of protein crystals is the stability that the PIL medium bestows on the folded protein. To assess this stability over the wide range of concentrations of this study we have chosen differential scanning calorimetry as a preferred characterization tool since the common spectroscopic tools for structural characterization, FTIR and circular dichroism, encounter problems with the very high concentrations of special interest in our work. DSC, as we have shown elsewhere [25] is a very discerning technique. When it can be shown that a protein in solution remains unchanged after the severe stress of denaturation and renaturation, then the stability of the protein in the solution during an ambient temperature crystal preparation process is well assured.

A subsidiary benefit of the DSC characterization is the information provided on the cooperative nature of the process by which the protein to be crystallized unfolds. According to Privalov [24] the process can be described as cooperative, in the limit of being a simple two-state (“all-or-nothing”) process, if the enthalpy of unfolding obtained by integration of the DSC endotherm yields the same value as that obtained from the endotherm peak value, using an equation derived from the van’t Hoff equation for temperature dependence of chemical reactions. We have illustrated the application of this relation to lysozyme unfolding in protic ionic liquids, elsewhere [25]. Strong deviations from the van’t Hoff-based unfolding enthalpy have implications for the structure of the protein dissolved within a given environment (solvent). In the limit, distinct structural states of the protein visited during unfolding and refolding, are distinguishable [25], and if these can be crystallized separately by our techniques then major advances could be made. The crystallization of partially unfolded states has not been sought in the present study, and remains for future evaluation.

**Figure 2:**
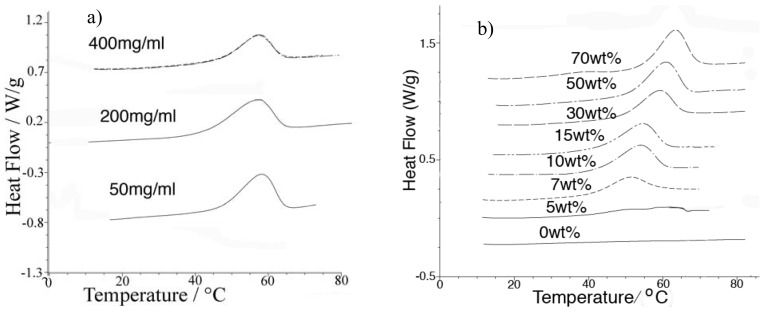
a) Two successive DSC thermograms of HEWL dissolved at 400 mg/mL in 25:75 EAN-water showing that no protein is lost when the protein unfolds in the present medium. The lower traces for lower concentrations of protein, (after scaling to constant total protein) show that the unfolding enthalpy does not depend on protein concentration; b) DSC thermograms of HEWL in a succession of EAN/water mixtures of increasing water content. All scans were conducted at 10 K/min.

Figure 2a (upper traces) shows successive DSC traces of HEWL at 400 mg/mL in the 75:25 EAN-water (by wt) mixture. The lower curves for sample protein concentrations of 50 mg/mL and 200 mg/mL scaled up appropriately to compensate for the different protein masses, show that the unfolding enthalpy is independent of the protein concentration. That HEWL can be refolded in high yield, even at these high concentrations, is demonstrated by the repeat DSC scan, which superimposes faithfully on the first trace —A surprising result given the remarkably high protein concentration of the study. Evidently the hydrated ionic liquid environment is very effective in inhibiting aggregation of the protein. This is of course consistent with the long storage life found [24] for protein solutions that exhibit high refolding indexes [26].

Figure 2b shows the DSC traces of the thermal unfolding of HEWL in various EAN/water mixtures, (wt % water) at the maximum HEWL solubility for each solvent composition as it appears in Figure 1, wt % scale. The thermal stability of the protein at each water content is indicated by the value of the denaturation temperature: we have not carried out the refoldability test in these cases. Except for the lowest water contents, the endotherms all indicate two-state cooperative refolding. The absence of any endotherm at all in the case of anhydrous EAN shows that HEWL is denatured in the absence of water, though (as shown elsewhere [27]), it can refold into the bioactive form on dilution to biological concentrations. With 5 wt % of water present, a very small endotherm (of shape incompatible with cooperative unfolding according to the van’t Hoff evaluation), is seen. By 10 wt % water, an endotherm of the two-state cooperative shape is observed, and this shape is retained at all other solvent compositions.

The denaturation temperature T_d_ increases markedly with increasing water content. The stability against aggregation, on the other hand, is greatest in the concentration range 15–30 wt % water, so thermal stability and resistance to aggregation seem to be distinguishable properties. It is well known that stability against aggregation is also strongly dependent on pH in solutions of the protein concentration that are normally studied (low concentrations by the standards of the present work). Since pH is not well defined in solutions of the high salt concentration encompassed in our study (no water structure survives when two of every three particles is an ion), we cannot be sure what role proton activity plays in the observed stability changes. At fixed water content, proton activity (which can be monitored by N-^1^H chemical shifts) [25] seems to affect T_d_ and the aggregation/misfolding propensity in the same way. However this proton activity monitor is unreliable for increasing water contents because of exchange of OH and NH protons. The mean pK_a_ of the acid and base making EAN is 4.7 so the increasing T_d_ may reflect decreasing acidity.

### 2.3. Formation of protein crystals from concentrated hydrated EAN solutions

In this section we document how the high protein concentration domain of EAN + water solutions can offer an alternative approach to producing protein crystals of good “X-ray quality”. Traditional protein crystallography relies on a dehydration procedure. Typically, a drop containing the protein in a suitable solution is exposed to a second solution containing a dehydrating additive, often polyethylene glycol or sodium chloride [7,8]. Ionic liquids have also been shown to be very effective as additives in these traditional protein crystallization methods [12,13,14,15]. It can be seen from Figure 1, that a reverse procedure, a “rehydration” procedure, is also possible by taking advantage of the minimum in the solubility of protein that precedes the ultra-high solubility domain in the PIL-water system. In a successful trial of this new approach, we have used as starting solution the solvent composition 57:33 (wt %) EAN-H_2_O containing HEWL at 100 mg/mL, and will report the findings below. Since the quality of the crystal obtained in this initial experiment is apparently rather good, it seems important to show first that the method can be expanded to include a variety of ionic liquid components.

**Figure 3:**
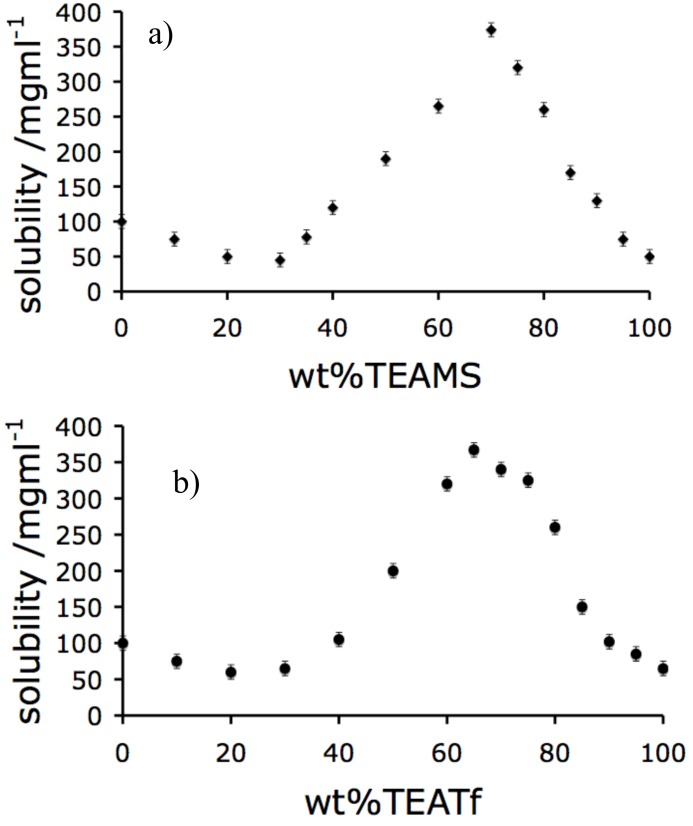
Solubility curves for Hen lysozyme, HEWL, dissolved in a) TEAMS and b) TEATf.

Figure 3 shows the equivalent of Figure 1 for two additional cases in which the EAN of the Figure 1 system is replaced by trimethylammonium methanesulfonate (TEAMS) in one case and by triethylammonium triflate (TEATf) in a second. Both have been components in previous studies of proteins dissolved in hydrated ionic liquids reported by this laboratory [26]. The HEWL solubility curves of all three systems are similar, with minimum solubilities near 75 wt % water and maximum values near 30 wt % water. We have established by proton magnetic resonance studies [25] that these solutions have proton activities (effective pHs determined by the state of the proton transferred in PIL formation) that differ from that of the equivalent EAN solution. The effective acidity goes as the mean of the pK_a_s of the acid and base that generate the PIL. Accordingly, TEAMS and TEATf will optimally stabilize proteins that, in aqueous solution, are known to be more acidophilic than lysozyme.

**Figure 4:**
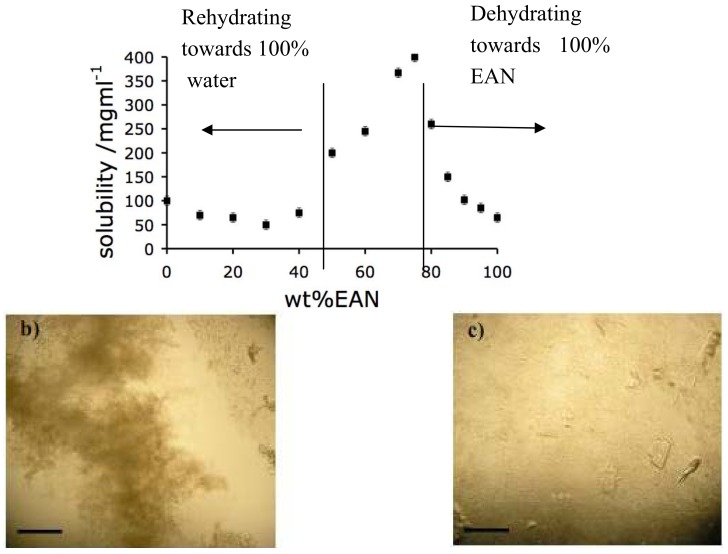
a) Solubility curve for HEWL dissolved in EAN/water solutions, illustrating the two approaches (“rehydration” and “dehydration”) to protein crystallization that have been tested in the present work. b) Optical micrograph of aggregated protein resulting from the use of NaCl as the precipitating additive. c) Optical micrograph of the product of the dehydration crystallization method when PEG is used as the precipitating additive: both small protein crystals and aggregated protein are in evidence.

The protein crystallization procedures tested on the basis of the solubility functions illustrated in Figure 1 and Figure 3, are illustrated in Figure 4. Both are distinct from the conventional approach, but only the rehydration procedure has produced useful results. The procedures are described in more detail in the Experimental Methods section, below. In the case of the dehydration procedure, the precipitation process was rapid, but only aggregates, or collections of small crystals of poor x-ray quality, were obtained, as exhibited in Figure 4b and c.

**Figure 5:**
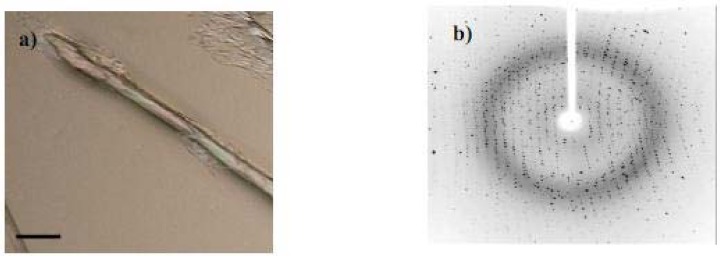
(a) Optical micrograph of a HEWL crystal produced by the rehydration method (b) X-ray diffraction pattern obtained for the crystal shown in (a). Scale bar is 50 μm.

However, using the rehydration strategy, a large crystal, the micrograph of which is shown in Figure 5a, slowly formed. This crystal proved to be of rather high X-ray quality, yielding the sharp Laue pattern of Figure 5b.

The pattern of Figure 5b was analyzed to a tetragonal unit cell, space group P4_3_2_1_2, with unit cell parameters a = b = 79.28 and c = 38.73 Å with resolution of 1.9 Å, consistent with literature values for the unit cell of HEWL [14,28], c.f. a = b = 79.054, c = 38.027 Å in ref [14]. This is not necessarily expected in view of the fact that the liquid filling the space between protein molecules is probably not water but rather an EAN-water solution. We note that the crystal of ref. [14] was obtained by the conventional approach but using EAN as a nucleating agent. The parameters for tetragonal lysozyme crystals grown in zero gravity by the counter-diffusion method [29] yield the parameters a = 77.06 and c = 37.22Å with very high resolution. In view of the fact that our procedures (see next section) have by no means yet been optimized, the rehydration approach to protein crystallization, using hydrated ionic liquid host solutions, obviously warrants further development.

## 3. Experimental

### 3.1. Sample preparation

EAN: water solutions, of compositions indicated in the solubility diagram Figure 1, and unfolding enthalpy diagram Figure 2b, were prepared by weighing, using doubly distilled water and EAN prepared as described in earlier work [26]. For solutions containing HEWL, the HEWL was added after the EAN/water mixture had been prepared and allowed dissolve at ambient temperature. The HEWL was purchased from Sigma Aldrich L6876 as lyophilized powder and used without further purification.

### 3.2. DSC

DSC thermograms were obtained on ~5 mg samples containing ~2 mg HEWL are using either TA2020 or Mettler Toledo scanning calorimeters. The scan rate in all cases was 10 K/min.

### 3.3. Solubility

Solubility of HE WL was determined using dynamic light scattering on a Malvern high performance particle sizer (HPPS) to detect when a given preparation was a homogenous solution as opposed to a suspension. Solutions containing a fixed amount of protein, 200 mg/mL were prepared and protein, or additional solvent, (the corresponding EAN/water) was added, until the solution registered clear by DLS. A separate solution containing the HEWL concentration that corresponded to the maximum solubility, as determined above, was then prepared and left for 24 hours and re-measured to ensure that the solution was stable. It was observed, in water-rich solutions, that higher concentrations could be obtained but then precipitation of solid from an initially clear solution (presumably due to aggregation of non-native states) was rapid.

### 3.4. Growth of protein crystals

The familiar hanging drop setup was used to grow crystals of HEWL, using the novel solvent conditions of this study to provide supersaturated solutions according to each of the composition changes indicated in Figure 4a. The crystallization tests were carried out at ambient temperature using a standard 24 well hanging drop crystallization screening tray that was sealed with clear tape.

For the dehydration procedure (see Figure 4), a mother liquor containing either NaCl (60 mg/mL) or PEG-400 (100 mg/mL) was prepared. The hanging drop, in each case, contained protein at the concentration 100 mg/mL. One hundred mg/mL of HEWL was dissolved in 80:20 (wt %) EAN-H_2_O (see Figure 4a), the mother liquor (15 μL, containing either the NaCl or the PEG) was mixed with the HEWL PIL-water solution (15 μL). In the cases where NaCl was used as the precipitating agent, aggregation of the protein occurred rapidly to give the product shown in Figure 4b. A combination of aggregated protein and small crystals (see micrograph Figure 4c) resulted when PEG-4000 was used as the precipitating agent, however the X-ray quality of the crystals was generally poor.

For the rehydrating procedure, a drop of HEWL (20 μL, 100 mg/mL) in the 57:43 (wt %) EAN- H_2_O solution, was used. The reservoir contained only water. The transfer of water occurred through the vapor phase as usual, *but in the reverse direction from normal*, as the system seeks its minimum free energy at uniform composition. The crystallization rate was, not surprisingly, much slower in the rehydration procedure; the total time to grow the crystal with dimension of 70 μm by 200 μm seen in Figure 5a, was three days. No attempt has yet been made to seek optimum crystallization rates, though these could be manipulated using a more hydrophilic ionic liquid or changing the reservoir temperature to increase the rate of water uptake.

## 4. Conclusions

We have reported an alternative approach to protein crystallization made possible by the unusual solubility profile of HEWL observed in three different systems of (protic ionic liquid + water) in which solubilities up to 400 mg/mL can be demonstrated. Using a hanging drop strategy featuring a direction of water transfer that is opposite to that of conventional hanging drop techniques, we obtain large crystals of good X-ray quality in our initial experiments. Refinements in methodology may be expected to improve crystal quality even further. Since ionic liquids with hydrophobic, as well as hydrophilic character, can be prepared, it is reasonable to hope that this protein crystallization strategy can be developed into one of broad applicability.
